# Rapid test to assess the escape of SARS-CoV-2 variants of concern

**DOI:** 10.1126/sciadv.abl7682

**Published:** 2021-12-03

**Authors:** Jacob T. Heggestad, Rhett J. Britton, David S. Kinnamon, Simone A. Wall, Daniel Y. Joh, Angus M. Hucknall, Lyra B. Olson, Jack G. Anderson, Anna Mazur, Cameron R. Wolfe, Thomas H. Oguin, Bruce A. Sullenger, Thomas W. Burke, Bryan D. Kraft, Gregory D. Sempowski, Christopher W. Woods, Ashutosh Chilkoti

**Affiliations:** 1 Department of Biomedical Engineering, Pratt School of Engineering, Duke University, Durham, NC 27708, USA.; 2 Department of Pharmacology and Cancer Biology, Duke University School of Medicine, Durham, NC 27710, USA.; 3 Center for Applied Genomics and Precision Medicine, Department of Medicine, Duke University, Durham, NC 27710, USA.; 4 Division of Infectious Diseases, Department of Medicine, Duke University Medical Center, Durham, NC 27710, USA.; 5 Department of Medicine and the Duke Human Vaccine Institute, Duke University School of Medicine, Durham, NC 27710, USA.; 6 Department of Surgery, Duke University School of Medicine, Durham, NC 27710, USA.; 7 Division of Pulmonary, Allergy, and Critical Care Medicine, Department of Medicine, Duke University Medical Center, Durham, NC 27710, USA.

## Abstract

Severe acute respiratory syndrome coronavirus 2 (SARS-CoV-2) variants are concerning in the ongoing coronavirus disease 2019 (COVID-19) pandemic. Here, we developed a rapid test, termed CoVariant-SCAN, that detects neutralizing antibodies (nAbs) capable of blocking interactions between the angiotensin-converting enzyme 2 receptor and the spike protein of wild-type (WT) SARS-CoV-2 and three other variants: B.1.1.7, B.1.351, and P.1. Using CoVariant-SCAN, we assessed neutralization/blocking of monoclonal antibodies and plasma from COVID-19–positive and vaccinated individuals. For several monoclonal antibodies and most plasma samples, neutralization against B.1.351 and P.1 variants is diminished relative to WT, while B.1.1.7 is largely cross-neutralized. We also showed that we can rapidly adapt the platform to detect nAbs against an additional variant—B.1.617.2 (Delta)—without reengineering or reoptimizing the assay. Results using CoVariant-SCAN are consistent with live virus neutralization assays and demonstrate that this easy-to-deploy test could be used to rapidly assess nAb response against multiple SARS-CoV-2 variants.

## Teaser

A rapid test measures neutralizing antibodies against SARS-CoV-2 variants of concern in 1 hour.

## INTRODUCTION

Severe acute respiratory syndrome coronavirus 2 (SARS-CoV-2), which causes coronavirus disease 2019 (COVID-19), has led to a major health crisis with substantial mortality and socioeconomic consequences worldwide. SARS-CoV-2 is a single-stranded RNA virus with four structural proteins: nucleocapsid, membrane, envelope, and spike (S) (*1*). The S protein—composed of the S1 and S2 domains—is exposed on the viral coat of SARS-CoV-2 and plays an essential role in viral attachment, fusion, entry, and transmission (*2*). Specifically, the receptor binding domain (RBD) of S1 binds to the SARS-CoV-2 cellular receptor—angiotensin-converting enzyme-2 (ACE2), which mediates viral entry into cells (*2*). Because of its critical role in viral entry, the S protein serves as the basis for COVID-19 vaccines and as the target for antibody-based therapeutics (*3*).

Although coronaviruses have genetic proofreading mechanisms to maintain their genome (*4*), they are still prone to mutations that can alter viral replication, transmission, and recognition by the host immune response. Of particular concern are mutations within the RBD because of its important role in viral entry. Recent genetic epidemiological surveillance has identified emerging SARS-CoV-2 variants that are circulating globally. Variant B.1.1.7 (also known as Alpha variant) originated in the United Kingdom (*5*) and contains nine mutations in the S protein, including one within the RBD—N501Y. This mutation increases the binding affinity to ACE2 (*6*, *7*) and contributes to the increased transmissibility of B.1.1.7 (*8*–*10*). Fortunately, several studies have shown that convalescent and vaccinee sera effectively cross-neutralize B.1.1.7 with only a minimal decrease in potency (*11*–*14*). Conversely, variant B.1.351 (Beta), which originated in South Africa (*15*), and variant P.1 (Gamma), which originated in Brazil (*16*) and Japan (*17*), each harbor three mutations within the RBD—K417N (B.1.351)/K417T (P.1), E484K, and N501Y. Evidence is mounting that these two strains can evade neutralization by monoclonal antibody (mAb) therapies and are more resistant to neutralization by polyclonal antibodies resulting from natural infection or immunization (*12*–*14*, *18*–*21*). These variants of concern (VOCs), as well as newly emerging ones, such as the B.1.617 (Delta) lineage identified in India (*22*), pose new challenges in the effort to contain the spread of the virus.

Assays to detect anti–SARS-CoV-2 antibodies are an important tool to assess natural or vaccine-induced humoral response at the individual patient level and for epidemiological surveillance at the population level. While many antibody binding assays have been developed for COVID-19 serodiagnosis (*23*–*27*), these tests are unable to determine the specific fraction of antibodies that can potentially neutralize the SARS-CoV-2 virus and thus confer protection. The main approaches for detecting neutralizing antibodies (nAbs) are microneutralization assays or plaque reduction neutralization tests, which monitor functional neutralization of SARS-CoV-2 entry/replication in permissive cells via nAbs binding to the RBD (*28*–*30*). However, these assays are labor-intensive, costly, and require highly trained personnel working in biosafety level 3 facilities. Neutralization assays using vesicular stomatitis virus and lentivirus pseudotyped with SARS-CoV-2 S protein have also been reported (*31*, *32*). These assays can be performed in biosafety level 2 facilities; however, they still require live cells and >24 hours to carry out the assay. To circumvent the need for viruses, permissive cells, and level 2 or 3 containment facilities, enzyme-linked immunosorbent assay (ELISA)–type ACE2-RBD blocking assays have been developed that mimic the virus-host interaction and serve as a surrogate for antibody neutralization activity (*33*–*37*). Specifically, these assays measure the ability of nAbs to block interactions between ACE2 and purified RBD using a competitive binding inhibition format in an ELISA plate. Several studies have demonstrated that this assay format shows high correlation with conventional neutralization tests (*33*, *34*), and thus its readout can serve as a proxy for the protection conferred by antibodies. With more transmissible and virulent SARS-CoV-2 strains now circulating globally, there is an urgent need for a test that can measure nAbs against several VOCs simultaneously by an easily deployable rapid test. Such a test could be useful to study the impact of RBD mutations on neutralization, to monitor the efficacy of vaccines against circulating VOCs in low-resource settings, to identify individuals who may be susceptible to reinfection or breakthrough infections even after vaccination, and to identify patients with COVID-19 who may benefit from mAb therapies.

**Fig. 1.**
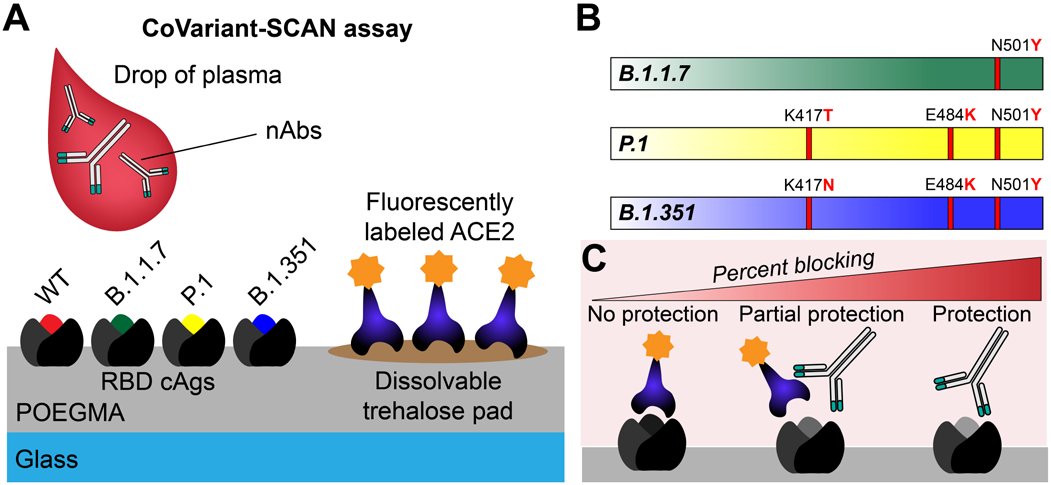
CoVariant-SCAN assay schematic. (**A**) WT, B.1.1.7, P.1, and B.1.351 RBD capture antigens (cAgs) are inkjet-printed onto a POEGMA surface. Nearby, fluorescently labeled ACE2 is inkjet-printed onto a dissolvable trehalose pad. When a plasma or serum sample is added to a chip, the trehalose pad dissolves, liberating the ACE2 from the surface, which diffuses across the surface. If a sample does not contain nAbs, ACE2 binds to the RBD cAgs, leading to a high fluorescence signal. If a sample contains nAbs, the nAbs block the ACE2-RBD binding interaction, leading to a lower fluorescence signal. (**B**) SARS-CoV-2 variant RBD mutations. Variant B.1.1.7 contains one RBD mutation: N501Y. Variants P.1 and B.1.351 each contain three RBD mutations: K417T (P.1)/K417N (B.1.351), E484K, and N501Y. (**C**) nAbs interfere with the RBD-ACE2 binding interaction to varying degrees. Greater blocking of this interaction indicates greater antibody neutralization.

To address this need, we report here a rapid test, termed the CoVariant-SCAN (COVID-19 Variant S-ACE2–Competitive Antibody Neutralization) assay, that evaluates the ability of host nAbs to block the pathologic interaction between variants of viral RBD and human ACE2 within 1 hour from a drop of plasma (Fig. 1A). As proof of principle, we demonstrate the performance of our assay against four SARS-CoV-2 strains—wild type (WT), B.1.1.7, P.1, and B.1.351. This assay is constructed by inkjet-printing RBD proteins from each variant (Fig. 1B) onto a “nonfouling” poly(oligoethylene glycol methyl ether methacrylate) (POEGMA) coating, as described previously (*27*, *38*). Nearby, fluorescently labeled human ACE2 is inkjet-printed upon a dissolvable trehalose pad (fig. S1 shows the chip layout). When a sample without nAbs is added, fluorescently labeled ACE2 dissolves from the POEMGA brush and binds to RBD capture sites, leading to a high fluorescence signal. In the presence of potential nAbs, the RBD-ACE2 interaction can be partially or completely blocked, resulting in a decrease in fluorescence signal (Fig. 1C). We demonstrate the multiplexing capability of CoVariant-SCAN by simultaneously assessing the neutralizing activity against WT, B.1.1.7, P.1, and B.1.351 from a single sample. We used CoVariant-SCAN to assess the efficacy of known neutralizing therapeutic mAbs, natural immunity from convalescent plasma, and vaccine-induced immunity. During this study, as the B.1.617.2 (Delta) variant emerged in India, we rapidly incorporated this variant into the CoVariant-SCAN assay without needing to reoptimize the assay, which shows that new VOCs can be easily accommodated, as they emerge.

## RESULTS

### Neutralization by monoclonal antibodies

We first assessed the potency of 28 mAbs against SARS-CoV-2 variants using the CoVariant-SCAN (table S1). These included 20 mAbs derived from a convalescent individual, 6 recombinant mAbs, and 2 mAbs with Emergency Use Authorization (EUA): REGN10987 (imdevimab) and REGN10933 (casirivimab). The convalescent donor-derived mAbs were isolated from plasmablasts or reactive memory B cells from a SARS-CoV-2–infected individual 11, 15, and 36 days after symptom onset, as previously described (*39*–*41*). Recombinant mAbs—purchased commercially—were isolated from immunized mice (*n* = 3) or rabbits (*n* = 2) or from a SARS-CoV-2–infected patient (*n* = 1). Last, the Regeneron mAbs were isolated from humanized mice and recovered patients, as described previously (*42*). All mAbs assayed on CoVariant-SCAN are specific to RBD. Each mAb was spiked into pooled human serum (PHS) collected before the COVID-19 outbreak at a starting concentration of 30 μg ml^−1^, and a dilution series spanning three logs was evaluated on CoVariant-SCAN chips. In parallel, the 20 convalescent patient-derived mAbs were characterized using a live virus microneutralization assay to benchmark the CoVariant-SCAN assay. For the CoVariant-SCAN assay, we defined the potency of each mAb by two metrics: (i) the percentage of ACE2/RBD binding blocked at the highest concentration that we assayed (30 μg ml^−1^) and (ii) the mAb concentration that blocks at least 20% of ACE2 binding to the target RBD. The first definition was chosen to mimic how CoVariant-SCAN could be used at the point of care to assay undiluted samples. Conversely, the second definition more closely resembles a traditional inhibitory concentration measurement, which requires testing a sample at multiple dilutions.

**Fig. 2.**
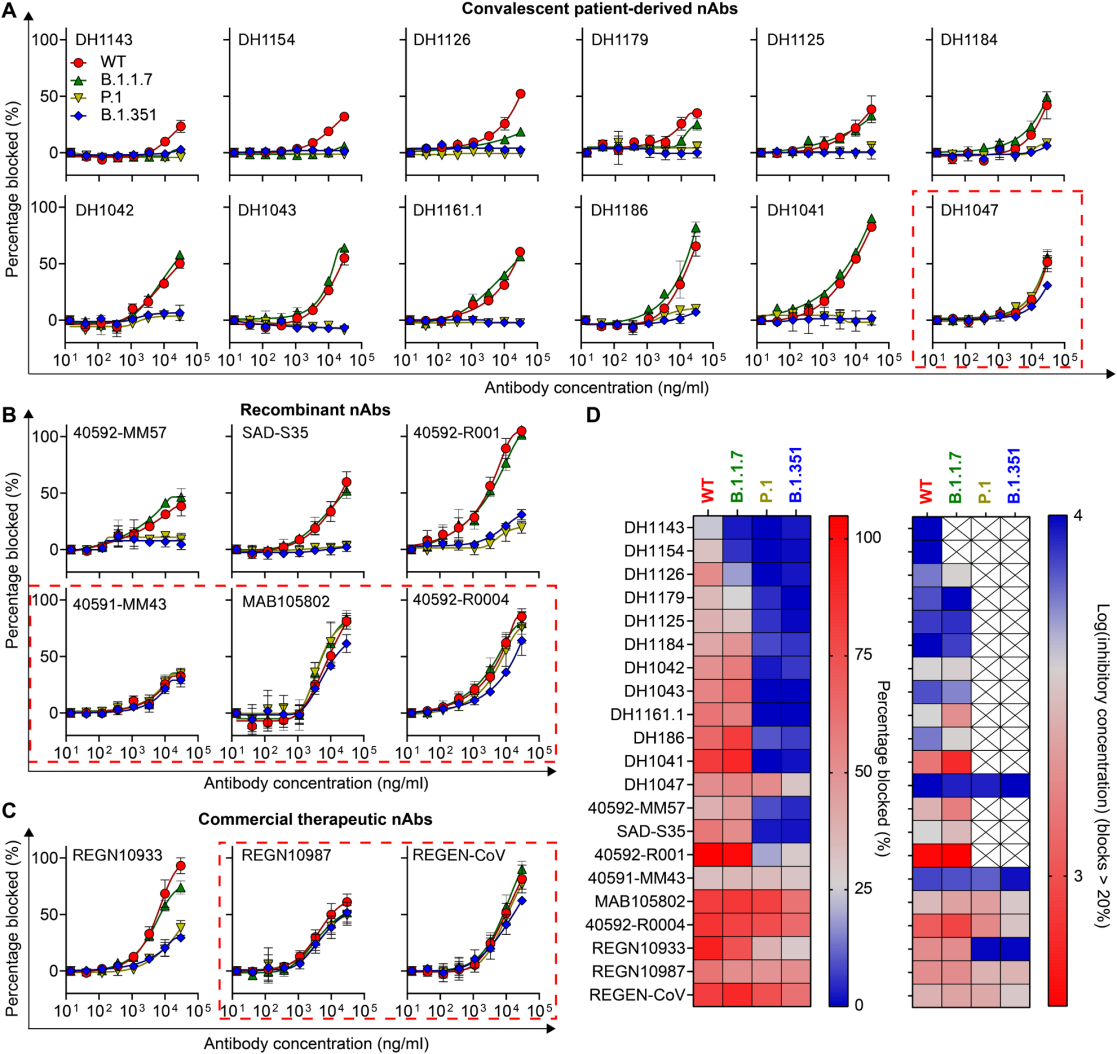
CoVariant-SCAN to assess potency of patient-derived, recombinant, and EUA-approved therapeutic mAbs. (**A**) Percentage of RBD-ACE2 binding blocked by 12 convalescent patient-derived mAbs against four RBD variants (WT, B.1.1.7, P.1, and B.1.351) as measured by the CoVariant-SCAN. Each mAb was spiked into prepandemic PHS, and a seven-point dilution series (30 μg/ml high dose, 1:3 dilutions) was tested in duplicate. Each dose was incubated for 1 hour and was then imaged on a GenePix scanner. Furthest left data points are blanks. (**B**) Percentage of RBD-ACE2 binding blocked by six commercially purchased recombinant nAbs. Seven-point dilution series tested in the same fashion as (A) but in triplicate. (**C**) Percentage of RBD-ACE2 binding blocked by Regeneron therapeutic antibodies. Antibodies were tested individually (REGN10933 and REGN10987) and together in the therapeutic cocktail (REGEN-CoV). Each data point represents the average of three independent assays. (**D**) Percentage of binding blocked at the highest dose (30 μg/ml) for each variant (left) and log-transformed inhibitory concentration that blocks >20% binding for each variant (right). Antibodies that do not block >20% of ACE2 binding are marked with an “X.” For both definitions, more potent antibodies are darker red. Antibodies that block all variants similarly are outlined with red dashes in (A) to (C).

We found that 12 of 20 mAbs derived from convalescent individuals had neutralizing activity in the WT live virus assay. These 12 antibodies also blocked ACE2 binding to WT RBD in a dose-dependent manner in the CoVariant-SCAN assay (Fig. 2A). The eight mAbs that were nonneutralizing in the live virus assay demonstrated weak or no blocking activity in CoVariant-SCAN despite having binding specificity to RBD, suggesting that our assay is specific to nAbs (fig. S2). In addition, all 20 convalescent patient-derived mAbs showed similar dose-response behavior when tested by an indirect assay, suggesting that their binding epitope—rather than their affinity for the RBD—is responsible for the differences in neutralizing/blocking activity (fig. S3). We found good concordance between the potency measured on the CoVariant-SCAN, compared to the WT live virus 50% inhibitory concentration (IC_50_), indicating that our test can reliably assess nAb activity (fig. S4). In addition, all six recombinant mAbs (Fig. 2B) and both EUA-approved mAbs (Fig. 2C) demonstrated dose-dependent blocking of ACE2 binding to WT RBD.

In general, the mAbs we assayed fell into one of two categories: (i) those that neutralize WT and B.1.1.7 similarly and have low or negligible potency toward P.1. and B.1.351 and (ii) those with similar potency across all variants. The first category consists of mAbs that likely target the ACE2-binding site within RBD, termed the receptor-binding motif (RBM) (*43*). Numerous studies have demonstrated that mutations that occur within the RBM drastically affect neutralization for antibodies targeting the RBM. Of particular concern are mutations at residue E484—as is the case for B.1.351 and P.1—which have a large effect on plasma antibody binding and neutralization (*44*–*47*). Notably, REGN10933 (*42*), which binds to the RBM, has been shown to have diminished neutralization toward B.1.351 (*14*) and P.1 variants (*48*, *49*), which is consistent with the results from CoVariant-SCAN. The second category contains mAbs that effectively blocked ACE2 binding to all variants similarly (enclosed in red dashed outlines). These mAbs likely target the “inner side” or the “outer side” of the RBD, as nAbs targeting these regions have been shown to retain neutralization activities against B.1.351 (*14*) and P.1 variants (*48*, *49*). Of note, REGN10987 (*42*), which targets the side of RBD, has been shown to retain its neutralization activity toward B.1.351 and P.1 variants and was able to block ACE2 binding to each RBD variant in CoVariant-SCAN. When both EUA-approved mAbs were tested as a cocktail (REGEN-CoV), WT, B.1.1.7, P.1, and B.1.351 variants were all neutralized similarly and effectively. The potency of each mAb toward all variants is summarized in Fig. 2D. Collectively, these results suggest that CoVariant-SCAN can be used to screen potential mAb therapeutics against SARS-CoV-2 variants and can identify mAbs with broad potency or that act synergistically in mAb cocktails. Notably, an interesting finding that emerges from a comparison of the Regeneron antibody cocktail with the other 26 mAbs clearly shows that compared to the rest of the mAbs tested in this study, the Regeneron cocktail offers robust protection against all three variants that is comparable to the protection conferred against WT SARS-CoV-2—against which these antibody drugs were originally developed.

### Neutralization by SARS-CoV-2–infected individuals

**Fig. 3.**
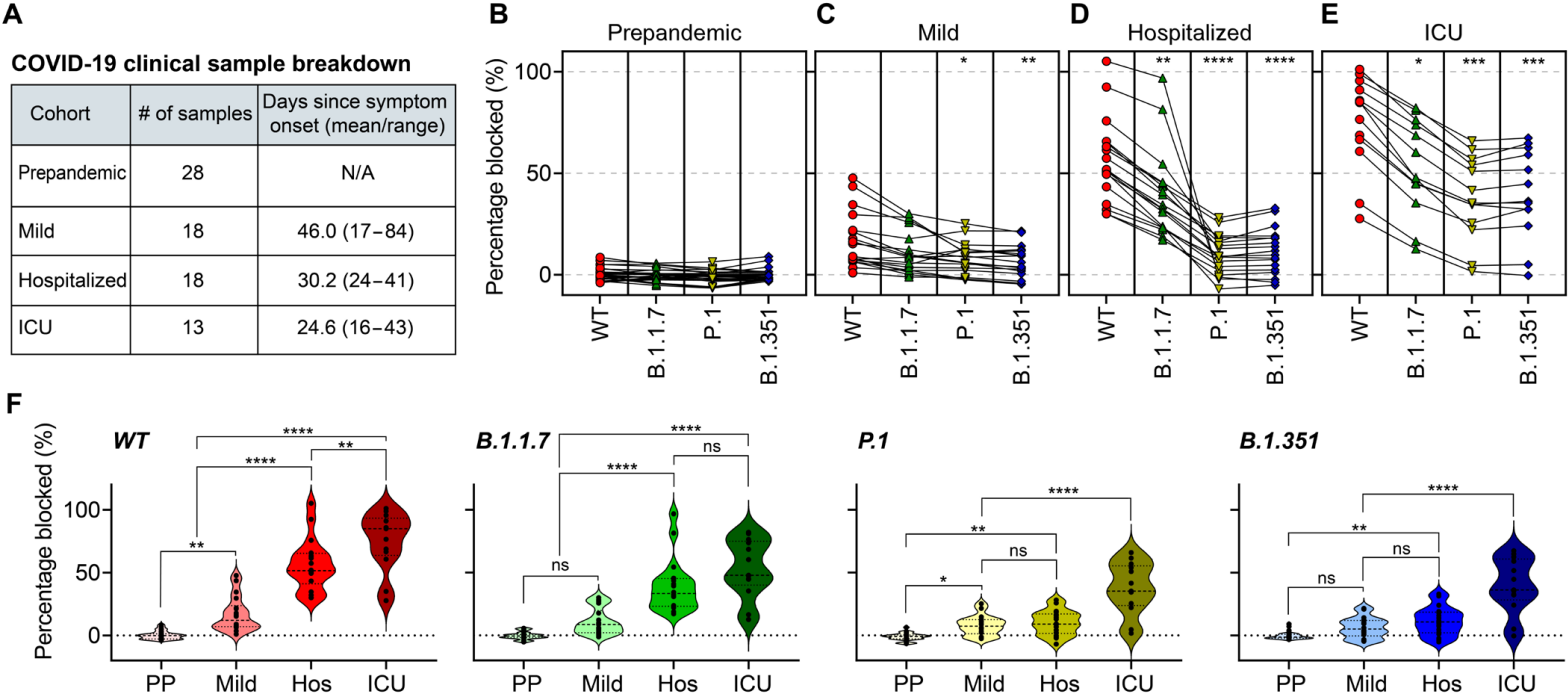
CoVariant-SCAN to assess natural humoral immunity. (**A**) Plasma samples were collected from four cohorts: prepandemic COVID-19 (−) individuals, mildly symptomatic patients with COVID-19 (+), hospitalized patients with COVID-19 (+) not requiring ICU admission, and patients with COVID-19 (+) admitted to the ICU. The table provides information on the number of samples and time of collection. (**B** to **E**) Percent blocking of RBD-ACE2 binding by patient plasma as measured by CoVariant-SCAN. Data are divided by patient cohort, where lines connect data from the same patient. Asterisks indicate significant difference from WT (* indicates adjusted *P* < 0.05, ** indicates adjusted *P* < 0.01, *** indicates adjusted *P* < 0.001, and **** indicates adjusted *P* < 0.0001) based on Dunnett’s multiple comparisons test. (**F**) Same dataset split by RBD variant. PP, prepandemic; Hos, hospitalized. Asterisks indicate a significant difference in percentage blocked between the marked cohorts using one-way ANOVA and Tukey’s multiple comparison post hoc test. All points shown are the average of two replicates.

We next assayed plasma from COVID-19–positive patients by CoVariant-SCAN. We obtained plasma from 13 patients with severe presentation who required admission to an intensive care unit (ICU), 18 patients with moderate presentation who required hospitalization but not admission to an ICU, 18 patients with mild presentation who did not require hospitalization or have worsening symptoms, and 28 prepandemic healthy negative controls. Individuals in the mild cohort exhibited one or more symptoms of COVID-19 (e.g., fever, cough, sore throat, malaise, headache, muscle pain, nausea, vomiting, diarrhea, and loss of taste and smell) but did not experience shortness of breath, dyspnea, or abnormal chest imaging. All positive samples were collected at least 2 weeks after symptom onset once seroconversion is expected to have occurred, with a mean of 46.0 days for the mild cohort, 30.2 days for the hospitalized/moderate cohort, and 24.9 days for the ICU cohort (Fig. 3A). All COVID-19–positive samples were collected before January 2021 before SARS-CoV-2 variants were widely circulating in the United States (*50*). For all samples, we used the percentage of ACE2-RBD blocked from undiluted samples as a proxy for antibody neutralization.

All 28 prepandemic negative controls showed negligible ACE2 blocking against each RBD variant (Fig. 3B). In the mild cohort, we identified several patients who developed nAbs against WT (Fig. 3C). There was a statistically significant difference in ACE2 blocking between all variants, as quantified by CoVariant-SCAN and determined by one-way analysis of variance [ANOVA; *F*(3,68) = 3.75, *P* = 0.0149]. Multiple comparisons by Dunnett’s test revealed that the WT group exhibited a statistically significant higher percent blocking compared to both P.1 (*P* = 0.0283) and B.1.351 (*P* = 0.0078) groups, indicating that neutralization against P.1 and B.1.351 was diminished relative to WT. Conversely, there was no significant decrease in neutralization against B.1.1.7 (*P* = 0.2068), indicating that B.1.1.7 can be cross-neutralized by convalescent plasma with only a modest decrease in potency, which is consistent with other studies (*12*, *51*). The mean fold decrease in percent ACE2 binding relative to WT was 1.5-fold for B.1.1.7, 2.2-fold for P.1, and 2.7-fold for B.1.351 for the mild illness cohort. For the hospitalized cohort, there was a statistically significant difference between each variant on CoVariant-SCAN (Fig. 3D), as determined by one-way ANOVA [*F*(3,68) = 33.41, *P* < 0.0001]. The percent blocking against WT was significantly higher compared to B.1.1.7 (*P* = 0.0082), P.1 (*P* < 0.0001), and B.1.351 (*P* < 0.0001), with a mean fold decrease (relative to WT) of 1.4-fold, 5.6-fold, and 4.9-fold, respectively. For the ICU cohort, there was a statistically significant difference in ACE2 blocking between each variant (Fig. 3E), as determined by one-way ANOVA [*F*(3,52) = 14.7, *P* < 0.0001]. Similar to the hospitalized cohort, blocking in the WT group was significantly higher than B.1.1.7 (*P* = 0.0383), P.1 (*P* = 0.0002), and B.1.351 (*P* = 0.0005). The mean fold decrease in percent ACE2 binding relative to WT was 1.4-fold for B.1.1.7, 2.0-fold for P.1, and 1.9-fold for B.1.351 in the ICU cohort.

Next, we compared the nAb blocking for each variant across the four sample cohorts (Fig. 3F). For all variants, there was statistically significant higher blocking for ICU samples compared to prepandemic controls (*P* < 0.0001) and the mild cohort (*P* < 0.0001). This is consistent with observations from other studies that severely ill patients generate higher titers of nAb compared to those with a mild infection (*35*, *51*, *52*). Hospitalized patients developed higher levels of nAbs against all variants compared to prepandemic controls; however, there was no significant difference between hospitalized and mild cases for P.1 and B.1.351 variants. We see a similar trend in the mild cohort as compared to the ICU and hospitalized cohorts, although the overall magnitude of the humoral response is blunted. Patients in the mild cohort had significantly higher nAb blocking against WT compared to prepandemic controls (*P* = 0.0028). For all other variants—B.1.1.7, P.1, and B.1.351—although some patients developed sufficient nAb levels to block ACE2 binding to all variants that were well above the baseline, only P.1 was statistically different when comparing mild infection versus prepandemic samples (*P* = 0.03).

Collectively, our results largely confirm the findings of studies that used live virus (*14*, *18*) or pseudovirus (*12*, *13*, *20*, *21*) neutralization assays in that convalescent plasma neutralizes B.1.1.7 similar to WT with only a modest decrease in potency, while activity against P.1 and B.1.351 is more severely diminished. In addition, individuals who developed more severe COVID-19 produced more nAbs with some degree of cross-neutralization against all VOCs tested. To directly test the concordance between CoVariant-SCAN and the gold standard microneutralization assay, a separate set of ICU samples that had been previously characterized by a live virus microneutralization assay were measured by CoVariant-SCAN (*27*). We found a strong correlation between the results of CoVariant-SCAN and the microneutralization assay, confirming the validity of our assay (fig. S5).

### Neutralization by vaccinated individuals

**Fig. 4.**
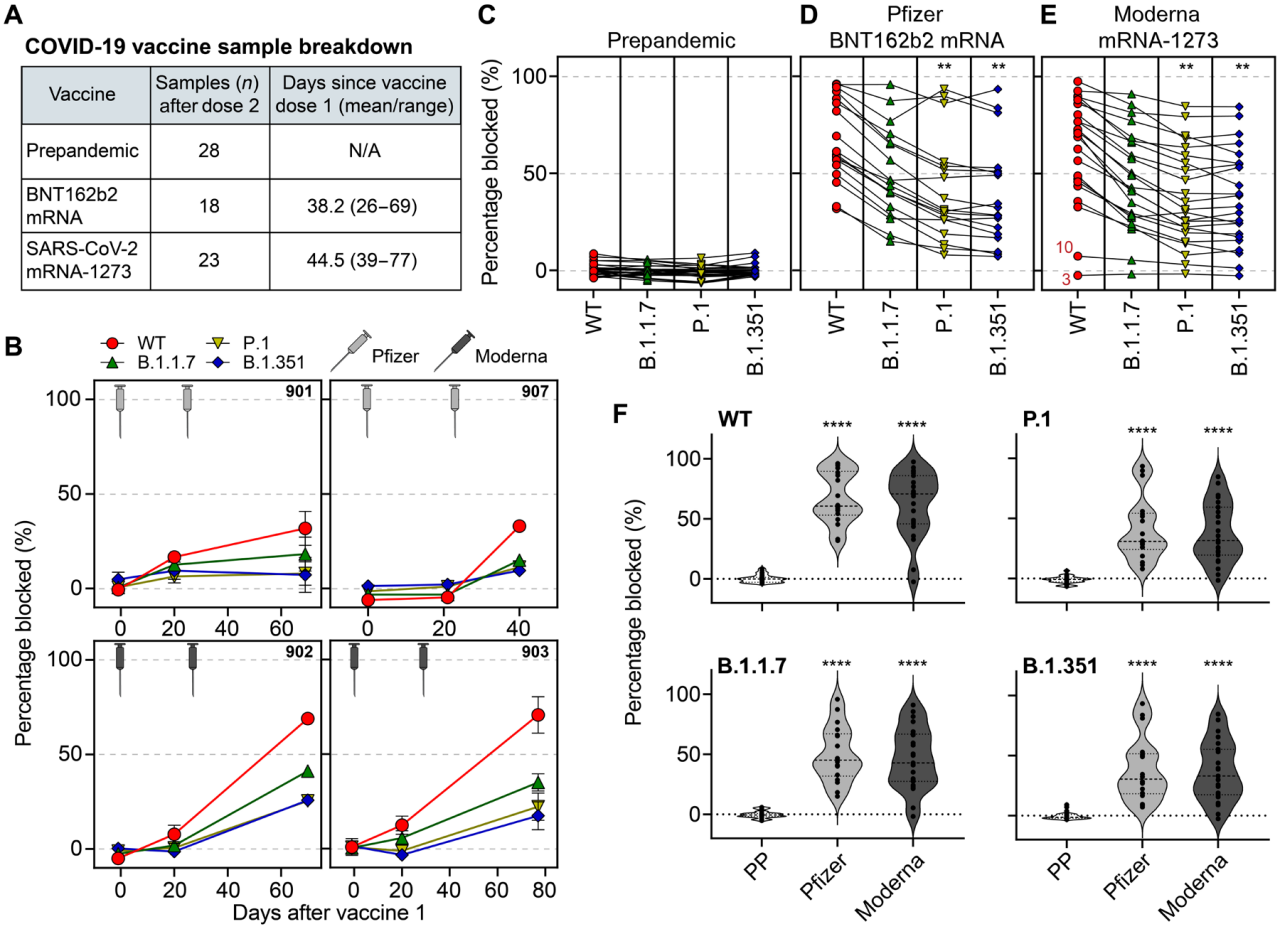
CoVariant-SCAN to assess vaccine-induced humoral immunity. (**A**) Plasma samples were collected from three cohorts: prepandemic (PP) healthy controls, individuals who received the Pfizer-BioNTech vaccine (BNT162b2 mRNA), and individuals who received the Moderna vaccine (SARS-Co-2 mRNA-1273). The data table provides information on the number of samples and time of collection. (**B**) Longitudinal data from two individuals who received the Pfizer vaccine (901 and 907) and two individuals who received the Moderna vaccine (902 and 903). Plasma samples from before dose 1, after dose 1, and after dose 2 were assayed on the CoVariant-SCAN in duplicate (SEM shown). (**C** to **E**) Percent blocking of RBD-ACE2 binding by patient plasma as measured by CoVariant-SCAN. Data are divided by vaccine cohort, where lines connect data from the same patient. Asterisks indicate significant difference from WT (** indicates adjusted *P* < 0.01) based on Dunnett’s multiple comparisons test. All points shown are the average of two replicates. Samples 10 and 3 in (E) were tested in an indirect assay (fig. 6) to determine whether anti-RBD–binding antibodies were present, despite low blocking activity. (**F**) Same dataset as (C) to (E) split by variant. For each variant, there was a significant difference in ACE2 blocking compared to prepandemic negative controls (**** indicates adjusted *P* < 0.0001) based on Tukey’s multiple comparisons test. There is no significant difference between the vaccine types for any RBD variant.

Next, we assayed plasma from COVID-19 vaccine recipients with CoVariant-SCAN. We tested plasma from 41 individuals, including individuals from whom longitudinal plasma samples were available (before first dose, after first dose, and after second dose). Of the 41 individuals, 18 received the BNT162b2 mRNA vaccine from Pfizer, and 23 received the mRNA-1273 vaccine from Moderna. The average days since receiving vaccine dose 1 was 38.2 for Pfizer and 44.5 for Moderna. Since dose 2, it was 18.1 and 28.0 days, respectively (Fig. 4A). Similar to the COVID-19–positive samples, we tested all plasma samples without processing or dilution, so we used the percentage of ACE2 blocked as a proxy for antibody neutralization.

We first tracked longitudinal samples from two individuals who received the Pfizer vaccine and two who received the Moderna vaccine at three time points during the immunization process: (i) prevaccine, (ii) >2 weeks after dose 1, and (iii) >2 weeks after dose 2 (Fig. 4B). We found that the CoVariant-SCAN could effectively track seroconversion in all four individuals and that all individuals developed nAbs that could block ACE2 binding to WT RBD, relative to their prevaccination control plasma sample. Antibody neutralization against B.1.1.7, P.1, and B.1.351 variants was attenuated relative to WT for all individuals, although it was still elevated compared to the prevaccine plasma samples. Notably, one dose of either Pfizer or Moderna vaccines did not yield sufficient nAb titers to block ACE2 binding against several variants, thus supporting the use of two-dose regimens to maximize neutralizing activity against WT and other VOCs.

Next, we examined neutralization against WT, and the B.1.1.7, P.1, and B.1.351 variants in individuals were sampled at least 1 week after their second dose. Prepandemic negative control samples (from Fig. 3B) are also shown in Fig. 4C as a baseline reference. We found that there was a statistically significant difference in neutralization against each variant in individuals receiving the Pfizer vaccine (Fig. 4D), as determined by one-way ANOVA [*F*(3,68) = 5.14, *P* = 0.0029]. ACE2 blocking by nAbs was significantly higher for WT compared to P.1 (*P* = 0.005) and B.1.351 (*P* = 0.002) variants, while there was no significant difference compared to B.1.1.7 (*P* = 0.0897). Similarly, in the Moderna cohort, neutralization was different across each variant (Fig. 4E), as determined by one-way ANOVA [*F*(3,88) = 5.02, *P* = 0.0029]. ACE2 blocking by nAbs was lower for P.1 (*P* = 0.004) and B.1.351 (*P* = 0.003) variants relative to WT, and there was no statistically significant difference compared to B.1.1.7 (*P* = 0.090). We found no significant difference in ACE2 blocking between the Pfizer and Moderna vaccines for any specific variant (Fig. 4F), and both vaccines significantly neutralized all variants tested relative to prepandemic negative control samples (adjusted *P* < 0.0005). We also tested plasma from three individuals who received the single-dose AD26.CoV2.S vaccine (codeveloped by Johnson & Johnson/Janssen), and the results are included in fig. S6; however, the sample size is too low to perform statistical analysis.

There was a relatively high amount of heterogeneity in the nAb response across all individuals tested. For example, some individuals developed nAbs that could block greater than 80% of ACE2 binding against all variants, while others (samples 3 and 10 in Fig. 4E) showed little to no neutralizing activity against any variant, despite developing anti-RBD antibodies when tested on an indirect assay that measures all immunoglobulin G (IgG) antibodies that bind to RBD (fig. S7). It is worth noting that the individuals with low nAb levels as measured by CoVariant-SCAN may still be protected from COVID-19 via mechanisms not directly related to ACE2-RBD blocking, which would require further investigation that is beyond the scope of this study. Overall, our results from CoVariant-SCAN are consistent with other studies (*19*) in that neutralization from vaccinee plasma against B.1.1.7 is essentially unchanged, while there is a significant loss in nAb activity against P.1 and B.1.351 variants, likely due to the E484K mutation (*44*–*47*). These findings may help explain COVID-19 breakthrough cases (*53*) that have occurred owing to infection with emerging variants even after immunization and support the continued development of variant-specific boosters (*54*).

### Demonstration of CoVariant-SCAN modularity

**Fig. 5.**
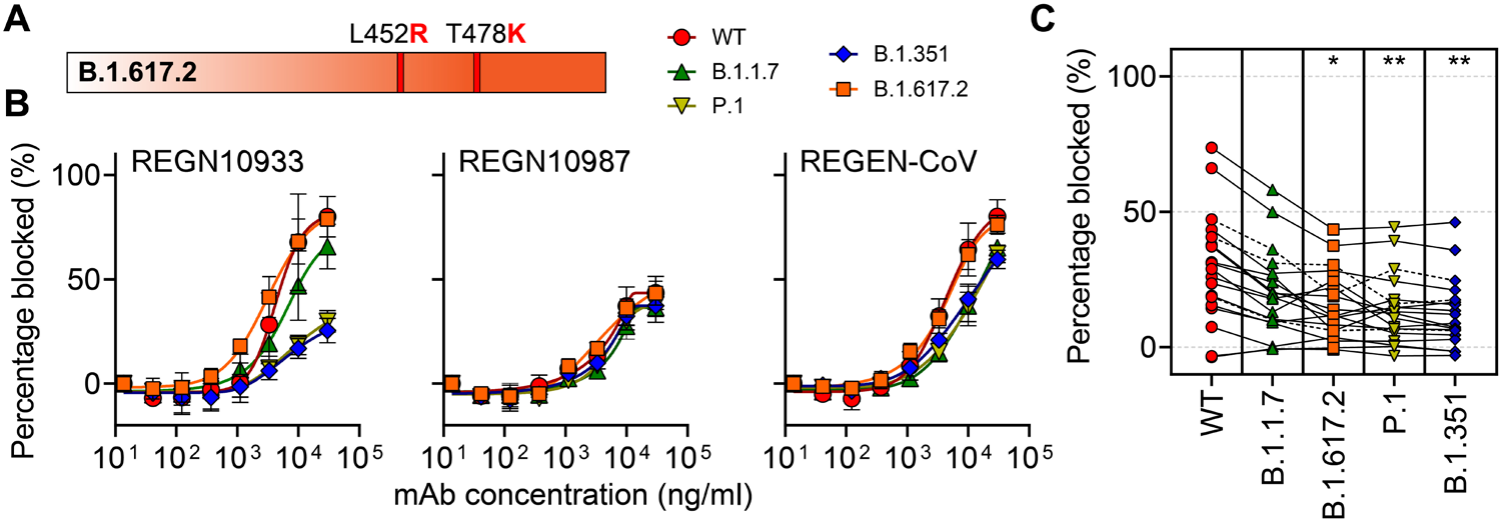
Modified CoVariant-SCAN to include B.1.617.2 (Delta) VOC. (**A**) The RBD protein for B.1.617.2, which includes two mutations—L452R and T478K—was incorporated into the CoVariant-SCAN assay. (**B**) Percentage of RBD-ACE2 binding blocked by Regeneron therapeutic antibodies. Antibodies were tested individually (REGN10933 and REGN10987) and together in the therapeutic cocktail (REGEN-CoV). Each data point represents the average of three independent assays. (**C**) Percent blocking of RBD-ACE2 binding by Pfizer (*n* = 12), Moderna (*n* = 4), and Johnson & Johnson (*n* = 3) vaccinee plasma as measured by the CoVariant-SCAN. Asterisks indicate significant difference from WT (* indicates adjusted *P* < 0.05, and ** indicates adjusted *P* < 0.01) based on Dunnett’s multiple comparisons test. All points shown are the average of two replicates. Dashed lines indicate that an individual had a previous confirmed COVID-19 diagnosis.

Last, to demonstrate the modular nature of the CoVariant-SCAN, we adapted the platform to detect nAbs against one additional VOC that was first identified in India in late 2020—B.1.617.2 (also known as the Delta variant)—which has since become the dominant circulating strain in many locations around the world (*55*). B.1.617.2 contains two mutations within the RBD: L452R and T478K (Fig. 5A). We first examined the potency of the Regeneron therapeutic mAbs on our assay against all VOCs. We found that REGN10933, REGN10987, and the cocktail of both mAbs remained active against the B.1.617.2 variant (Fig. 5B), which is consistent with previous studies (*56*). Next, we tested plasma from a new cohort of 19 individuals who received both doses of Pfizer (*n* = 12), Moderna (*n* = 4), or a single dose of the Johnson & Johnson vaccine (*n* = 3) (Fig. 5C). The mean time since the first dose for this cohort was 15.9 weeks. We found that there was a statistically significant difference in the percentage of ACE2-RBD blocking among the different VOCs as determined by a one-way ANOVA [*F*(4,90) = 3.725, *P* = 0.0075]. ACE2 blocking by nAbs was lower for B.1.617.2 (*P* = 0.026), P.1 (*P* = 0.009), and B.1.351 (*P* = 0.004) variants relative to WT, and there was no statistically significant difference for B.1.1.7 (*P* = 0.196) as determined by Dunnett’s multiple comparisons test. The data partitioned by vaccine type are shown in fig. S8. These findings are consistent with other studies (*56*) and suggest that the B.1.617.2 variant may be able to evade nAbs from vaccinee plasma; however, most individuals tested by our assay still developed some degree of neutralizing/blocking activity. This study highlights a key attribute of CoVariant-SCAN: the ability to rapidly incorporate additional RBD proteins from new VOCs as they emerge without the need to reoptimize the assay by simply adding another column of printed spots of the RBD for that VOC.

## DISCUSSION

The emergence of new SARS-CoV-2 variants with the potential to escape neutralization by therapeutic mAbs and natural or vaccine-induced immunity is of great concern. As sequencing programs continue to identify mutations within the SARS-CoV-2 genome, functional tests are needed to rapidly assess the impact of those mutations, especially those that arise in the S gene, due to its critical role in viral entry, propagation, and transmission. As of late 2020, several new variants of interest have been identified that are circulating in New York (*57*), California (*58*), India (*59*), and other locations around the world, all of which contain mutations in S. Monitoring the potential escape of these and future variants needs to be a priority to reduce the risk of resurgence in cases, even in highly seropositive populations, as SARS-CoV-2 will likely become endemic (*60*).

In this study, we developed a rapid test, termed the CoVariant-SCAN, to simultaneously assess the ability of antibodies to block the ACE2-RBD interaction against five SARS-CoV-2 variants: WT, B.1.1.7, P.1, B.1.351, and B.1.617.2. Our work is motivated by the urgent need for a rapid and easy-to-use assay that supplements conventional antibody neutralization tests that are labor-intensive, costly, require highly trained personnel, and are thus inaccessible in many regions around the world. While other assays have been developed that measure the ability of nAbs to block ACE2-RBD binding (*33*, *34*, *37*), to the best of our knowledge, CoVariant-SCAN is the first test that can detect nAbs against several SARS-CoV-2 variants simultaneously within 1 hour. Furthermore, because the CoVariant-SCAN is built upon a “nonfouling” polymer coating that eliminates nearly all nonspecific binding, the assay can be conducted directly from undiluted plasma (*38*, *61*, *62*). Although CoVariant-SCAN is not quite as sensitive as the cPass SARS-CoV-2 neutralization antibody detection kit (fig. S9)—developed by Lin-Fa Wang’s group (*33*) and commercialized by GenScript—our multiplexed assay only requires a single 1-hour incubation, which could be automated with a newly developed microfluidic chip (*27*) and read with a portable detector (*27*, *62*), making the platform well suited for point-of-care use in resource poor environments.

Using the CoVariant-SCAN, we demonstrated that several mAbs partially or completely lose blocking/neutralizing activity toward the RBD proteins of B.1.351 (K417N, E484K, and N501Y) and P.1 (K417T, E484K, and N501Y) variants. B.1.1.7, which only contains the N501Y mutation within the RBD, is less prone to escape neutralization by mAbs raised against WT RBD. This finding is consistent with other studies that used authentic virus and pseudovirus assays (*13*, *14*, *21*). We also demonstrated that CoVariant-SCAN specifically measures nAbs, as our assay measured weak or no blocking activity for a panel of eight convalescent patient-derived mAbs with binding specificity to RBD but no neutralizing capacity.

Our data confirm that the EUA-approved mAb REGN10933 may lose activity against B.1.351 and P.1, but when combined with REGN10987, blocking/neutralization is less severely affected by VOCs, highlighting the importance of identifying potent mAb cocktails and continued monitoring of their effectiveness against emerging VOCs. Comparison of the REGN antibody cocktail with the Pfizer and Moderna vaccines shows that the Regeneron therapeutic antibody cocktail possibly provides greater protection against the highly infectious Delta variant that is currently displacing other variants worldwide. Given that the individual response of vaccinated individuals against the Delta variant is, on average, up to 50% lower than WT, whereas neutralization of the Delta variant by the antibody cocktail is similar to WT, we suggest that this cocktail may be useful as a prophylactic for vaccinated individuals with a low level of nAbs against the Delta variant who are at increased imminent risk of exposure to this variant, such as because of travel to a region where that VOC is endemic.

We also investigated the ability of polyclonal antibodies from the plasma of naturally infected individuals to neutralize each SARS-CoV-2 variant. We found that individuals with mild, moderate, and severe cases of COVID-19 developed nAbs that could effectively block ACE2-RBD binding for the WT virus. Severe cases that required hospitalization in the ICU resulted in a more robust nAb response that could neutralize B.1.1.7, P.1, and B.1.351, albeit at a diminished level relative to WT. For mild and moderate cases, ACE2 blocking against P.1 and, B.1.351 variants was more similar compared to prepandemic plasma. These results suggest that individuals with mild or moderate COVID-19 infections may not produce sufficient nAb titers to prevent reinfection from P.1 or B.1.351 variants, which may explain the reinfection cases that have been appearing globally (*63*–*65*). These findings also underscore the importance of control measures to avoid resurgence in locations, even where seropositivity is already high, as is happening in Manaus, Brazil (*16*).

Next, we assessed the ability of CoVariant-SCAN to measure the neutralizing activity of vaccinee plasma. We found that individuals who received the Pfizer and Moderna vaccines developed nAbs that could neutralize all variants relative to prepandemic negative control plasma; however, activity against P.1 and B.1.351 variants were significantly attenuated relative to WT. Conversely, B.1.1.7 neutralization was relatively unaffected. We also demonstrated that the nAb titer continues to increase after the second dose of the Pfizer and Moderna vaccines and that the second dose yields nAbs that are better at cross-neutralizing VOCs. Our data also show that there is a considerable individual heterogeneity in nAb levels and that some individuals develop robust responses against all variants tested, while others may benefit from variant specific boosters that are currently being developed. The CoVariant-SCAN is an ideal platform to identify those individuals because it could be conducted at the point of care, is easily manufactured at scale, and can be deployed globally independent of a cold-chain or centralized testing laboratory.

Another key strength of our platform is the ability to rapidly test the impact of S protein mutations on immunity as they arise in newly emerging VOCs. Our workflow only requires inkjet printing purified RBD proteins as a row of separate capture sites without any changes to the detection reagent, making further multiplexing simple. As SARS-CoV-2 variant sequences are identified and deposited into repositoriessuch as the Global Initiative on Sharing Avian Influenza Data (GISAID) (*66*), recombinant RBDs from these variants can be quickly expressed, purified, and integrated into our assay, as we demonstrated with the B.1.617.2 variant, while this study was in progress. Although we focused on mutations within the RBD for this study, we can also use the full S1 protein—which contains additional mutations in VOCs—as the capture antigen on the CoVariant-SCAN (fig. S10). Likewise, we could prospectively assess the impact of modifications at important residues on immunity, as others have done (*46*, *47*), to identify mutations of concern. Therefore, we believe that the bespoke nature of CoVariant-SCAN will be useful to assess the impact of emerging SARS-CoV-2 mutations (*67*).

There are several potential scenarios where the CoVariant-SCAN could be useful. First, it could be deployed as an epidemiological tool to assess the efficacy of vaccines against circulating or emerging VOCs in specific regions. Second, it could be used to monitor individual patients’ risk for future infection by a circulating VOC based on their nAb profile. Similarly, the CoVariant-SCAN could be used at the bedside to test patients presenting with acute COVID-19 who are either known to have been infected by a VOC or if there is a high burden of VOC in their community, making it likely that their infection is caused by a VOC. Patients with low neutralizing activity could be treated immediately with the Regeneron cocktail or a similar mAb therapy to reduce the likelihood of severe infection. This approach would be especially useful for patients who are immunocompromised at the time of SARS-CoV-2 infection or vaccination, as they are likely to have a weaker humoral response and therefore are more at risk for reinfection and/or severe disease.

There are several limitations of the CoVariant-SCAN that warrant further discussion. First, our assay uses ACE2-RBD blocking as a proxy for antibody neutralization, which does not account for other antibody-mediated effector functions such as complement activation or antibody-dependent cellular cytotoxicity which can contribute to immunity (*68*). Furthermore, we only consider mutations within the RBD and therefore do not account for mutations occurring at other locations that could affect neutralization, such as within the N-terminal domain of S, which is an important target of several nAbs (*69*). Our assay format also does not consider the role of cellular immunity from memory T cells after primary infection or immunization, which is known to play an important role in preventing SARS-CoV-2 infection (*70*–*72*). Despite these limitations, we believe that CoVariant-SCAN is a promising rapid test to provide public health officials with the tools for effective serologic surveillance of SARS-CoV-2 variants and to answer questions being raised about the effectiveness of immunity—both natural and vaccine-induced—against emerging variants of SARS-CoV-2.

## MATERIALS AND METHODS

### Study design

The goal of this study was to develop a rapid test to assess the ability of nAbs to block ACE2 binding to SARS-CoV-2 RBD proteins from several SARS-CoV-2 VOCs. To do so, we first evaluated monoclonal antibodies with known neutralizing activity. For these monoclonal antibodies, our assay was benchmarked against a live virus microneutralization assay (Isolate USA-WA1/2020, NR-52281) to establish concordance between CoVariant-SCAN and the microneutralization assay using Pearson’s *r* correlation (conducted in GraphPad Prism). We also examined plasma from healthy controls, convalescent individuals with varying disease severity, and COVID-19 vaccine recipients. Sample sizes were chosen on the basis of availability of clinical samples in existing repositories or through commercial vendors. To examine the difference between ACE2-RBD blocking among different groups or different variants, one-way ANOVA was performed with post hoc testing (GraphPad Prism). A subset of convalescent samples had previously been characterized using the live virus neutralization assay, and the results from the CoVariant-SCAN were hence benchmarked using those samples. All experiments were performed in multiple replicates, as indicated throughout Materials and Methods and figure legends. As this was an observational study, experiments were not randomized or blinded; however, all clinical samples were identically tested and analyzed.

### CoVariant-SCAN assay fabrication

The CoVariant-SCAN assay builds upon the D4 immunoassay, described previously (*38*). Briefly, glass substrates were coated with a POEGMA polymer brush with a thickness of ~50 nm by surface-initiated atom transfer radical polymerization (SI-ATRP) (*73*). Next, recombinant SARS-CoV-2 RBD proteins for WT (Sino Biological, catalog no. 40592-V08H), B.1.1.7 (Sino Biological, catalog no. 40592-V08H82), P.1 (Sino Biological, catalog no. 40592-V08H86), and B.1.351 (Sino Biological, catalog no. 40592-V08H85) variants were immobilized on the POEGMA-coated glass slides using a Scienion S11 sciFLEXARRAYER (Scienion AG) inkjet printer. Columns of five ~180-μm-diameter capture spots for each RBD variant were printed at a concentration of 0.8 mg/ml (fig. S1). Surrounding the capture spots, 12 1-mm-diameter trehalose spots were printed using a BioDot AD1520 printer (BioDot Inc.) loaded with a 10% (w/v) trehalose solution (~100-nl drop volume). Next, Alexa Fluor 647–labeled human ACE2 (Sino Biological, catalog no. 10108-H05H) were deposited on top of the excipient pads using the BioDot printer at a concentration of 0.02 mg/ml. Twenty-four assays with this configuration were printed on each 75.6 mm by 25.0 mm by 1.0 mm glass slide in a 3 × 8 array. CoVariant-SCAN assays were stored under vacuum for at least 24 hours before use. For experiments with the B.1.617.2 variant, an additional column of five capture spots was inkjet-printed onto the POEGMA surface (Sino Biological, catalog no. 40592-V08H90).

### Analytical testing using the CoVariant-SCAN assay

CoVariant-SCAN chips were secured in a 96-well microarray hybridization cassette or adhered to a laser-cut acrylic that separates the chip into 24 separate wells. To perform the assay, 60 μl of sample was added directly to an assay well, covered, and incubated at room temperature for 1 hour. Although we chose 1 hour, we found that the incubation time does not drastically affect the results (fig. S11), and assays could be incubated for 20 min. After incubation, samples were aspirated, and chips were rinsed in wash buffer [0.1% Tween-20 in 1× phosphate-buffered saline (PBS)], dried, and then scanned with an Axon Genepix 4400 tabletop scanner (Molecular Devices LLC). PHS that was collected prepandemic was tested on each chip to serve as a negative control. The average fluorescence intensity at each capture spot was quantified using the Genepix Pro 7 analysis software. All fluorescence intensities were log-transformed before analysis. To calculate percentage blocked, we used the following formula

$$\%\;\text{Blocking}=100\times (1-\frac{x-B}{\text{NC}-B})$$

where *x* is the log-transformed intensity, *B* is a constant (2.301) representing the background fluorescence signal, and NC is the log-transformed intensity of the negative control samples, which was calculated separately for each experiment and for each variant.

All mAbs were diluted in PHS collected prepandemic. For experiments with recombinant and EUA-approved mAbs, each dose was run in triplicate. For experiments with the convalescent patient-derived mAbs, each dose was run in duplicate. We tested a seven-point dose-response curve for all mAbs with a starting concentration of 30 μg/ml. All data were plotted using GraphPad Prism version 9.1.1 (GraphPad Software). Regression analysis was performed in GraphPad using an asymmetric, five-parameter logistic equation for dose-response experiments.

All individual donor plasma samples (prepandemic healthy controls, mild, ICU, and vaccine recipients) assayed during this study were tested identically. Plasma samples were thawed from −80°C storage and allowed to reach room temperature before testing on the CoVariant-SCAN. Each sample was tested undiluted in duplicate to assess the reproducibility of the assay, where we found a strong correlation between replicates (fig. S12). The percentage blocked was calculated as described above and plotted as the mean of duplicate assays.

### Source of monoclonal nAbs

Convalescent donor-derived monoclonal antibodies isolated as previously described (*39*, *40*) were acquired from the Duke Human Vaccine Institute Pandemic Prevention Program under a material transfer agreement. The recombinant mAbs were purchased commercially (Sino Biological, catalog nos. 40591-MM43, 40592-MM57, 40592-R0004, and 40592-R001; ACRO Biosystems, catalog no. SAD-S35; R&D Systems, catalog no. MAB105802). Regeneron therapeutic mAbs were acquired from the Duke University Medical Center Pharmacy. All mAbs are summarized in table S1.

### Clinical samples

Deidentified plasma samples from severe COVID-19 cases requiring hospitalization in the ICU were accessed from the Duke COVID-19 ICU biorepository (Pro00101196, PI Bryan Kraft) approved by the Duke Health Institutional Review Board (IRB). For the mild and moderate/hospitalized cohorts, plasma samples from patients with confirmed SARS-CoV-2 infection were identified through the Duke University Health System or the Durham Veterans Affairs Health System and enrolled into the Molecular and Epidemiological Study of Suspected Infection (MESSI; Pro00100241). Samples were accessed via the same exempt protocol (Pro00105331, PI Ashutosh Chilkoti). Clinical severity was measured according to the National Institutes of Health clinical grading scale. Patients with severe infection have SpO_2_ <94% on room air at sea level, a ratio of arterial partial pressure of oxygen to fraction of inspired oxygen (PaO_2_/FiO_2_) <300 mm Hg, respiratory frequency >30 breaths/min, or lung infiltrates >50%. The moderate/hospitalized disease cohort was defined as individuals who experienced symptoms requiring hospitalization but did not require admission to the ICU. For four individuals, symptom onset was unknown; however, samples were taken at least 3 weeks after hospitalization. For calculation of the mean days since symptom onset (Fig. 3A), those samples were excluded. Those with mild disease may have any of the various signs and symptoms of COVID-19 (e.g., fever, cough, sore throat, malaise, headache, muscle pain, nausea, vomiting, diarrhea, loss of taste, and smell) but do not have shortness of breath, dyspnea, or abnormal chest imaging. Microneutralization assessments of clinical samples were run under Pro00105165 (PI Sempowski). Twenty-eight prepandemic negative control plasma samples were purchased commercially (Lee BioSolutions Inc. and Innovative Research Inc.). Plasma samples from vaccine recipients were either collected under the MESSI protocol or purchased commercially (Innovative Research Inc. and RayBiotech Life Inc.). For the experiments with the B.1.617.2 variant (Fig. 5C), fresh blood from 19 individuals was collected in EDTA-coated tubes under a Duke IRB protocol (Pro00106419, PI Woods). Blood was processed to plasma by centrifugation at 1800 relative centrifugal force for 15 min at 4°C. Plasma was aspirated from the top layer, aliquoted, and stored at −80°C before assaying on the CoVariant-SCAN. All samples were deidentified and tested under an exempt protocol (Pro00105331, PI Ashutosh Chilkoti). Each cohort is summarized in table S2.

### Indirect assay

To fabricate indirect assays to detect anti-RBD antibodies, SARS-CoV-2 WT RBD was inkjet-printed onto POEGMA-coated glass slides, as described above. Chips were placed in a microarray cassette to separate the chip into 24 separate arrays. Next, 60 μl of each sample was added to arrays in duplicate and incubated for 45 min. After incubation, each assay was washed three times with 100 μl of wash buffer (0.1% Tween-20 in 1× PBS), and then 60 μl of Alexa Fluor 647 fluorescently labeled mouse anti-human IgG (Southern Biotech, catalog no. 9040-01) at 2 μg/ml was added to each array for 15 min. Last, slides were washed, dried, and imaged on an Axon Genepix tabletop scanner. For experiments with nonneutralizing mAbs isolated from convalescent patients, each mAb was spiked into PHS diluted 1:10 [1% (w/v) bovine serum albumin and 0.05% Tween-20 in PBS diluent solution] at concentrations ranging from 4.1 ng/ml to 3 μg/ml. Dose-response curves were fit in GraphPad using an asymmetric, five-parameter logistic equation. For experiments with plasma samples from vaccine recipients and individual donor prepandemic healthy controls (Fig. 4), samples were diluted 1:10 [1% (w/v) bovine serum albumin and 0.05% Tween-20 in PBS diluent solution] and then assayed as described.

### Microneutralization assay

The SARS-CoV-2 virus (Isolate USA-WA1/2020, NR-52281) was deposited by the Centers for Disease Control and Prevention and obtained through the BEI (Biodefense and Emerging Infections) Resources Repository, National Institute of Allergy and Infectious Diseases, National Institutes of Health. SARS-CoV-2 microneutralization assays were adapted from a previous study as follows (*74*). Recombinant antibodies or plasma samples were diluted twofold and incubated with 100 TCID_50_ (median tissue culture infectious dose) virus for 1 hour. These dilutions were transferred to a 96-well plate containing 2 × 10^4^ Vero E6 cells per well. Following a 96-hour incubation, cells were fixed with 10% formalin, and cytopathic effect was determined after staining with 0.1% crystal violet. Each batch of microneutralization includes a known neutralizing control antibody (Sino Biological, catalog no. 40150-D001). Data are reported as IC_50_ or the inverse of the last concentration at which a test plasma protects Vero E6 cells.

### Statistical analysis

All statistical analysis was performed using GraphPad Prism version 9.1.1 (GraphPad Software Inc.). All data were log-transformed for analysis. Regression analysis was performed in GraphPad using an asymmetric, five-parameter logistic equation for dose-response experiments. One-way ANOVA was performed to establish statistical significance between different groups followed by post hoc multiple comparison tests (Tukey or Dunnett’s). Pearson’s *r* correlation was calculated to assess the degree of agreement between different assays and replicates.

## Notes

View/request a protocol for this paper from *Bio-protocol*.
