# Supplementary material for: Rapid test to assess the escape of SARS-CoV-2 variants of concern
Source: Sci Adv. 2021 Dec 3;7(49):eabl7682. doi: 10.1126/sciadv.abl7682 (PMC8641938; doi:10.1126/sciadv.abl7682)
Supplement: Supplementary file 1 — Figs. S1 to S12 Tables S1 and S2 Legend for data S1 [file sciadv.abl7682_sm.pdf]

Science Advances
AAAS

Supplementary Materials for

Rapid test to assess the escape of SARS-CoV-2 variants of concern

Jacob T. Heggestad, Rhett J. Britton, David S. Kinnamon, Simone A. Wall, Daniel Y. Joh, Angus M. Hucknall, Lyra B. Olson, Jack G. Anderson, Anna Mazur, Cameron R. Wolfe, Thomas H. Oguin III, Bruce A. Sullenger, Thomas W. Burke, Bryan D. Kraft, Gregory D. Sempowski, Christopher W. Woods, Ashutosh Chilkoti*

*Corresponding author. Email: chilkoti@duke.edu

Published 3 December 2021, *Sci. Adv.* **7**, eabl7682 (2021)
DOI: 10.1126/sciadv.abl7682

The PDF file includes:

Figs. S1 to S12
Tables S1 and S2
Legend for data S1

Other Supplementary Material for this manuscript includes the following:

Data S1

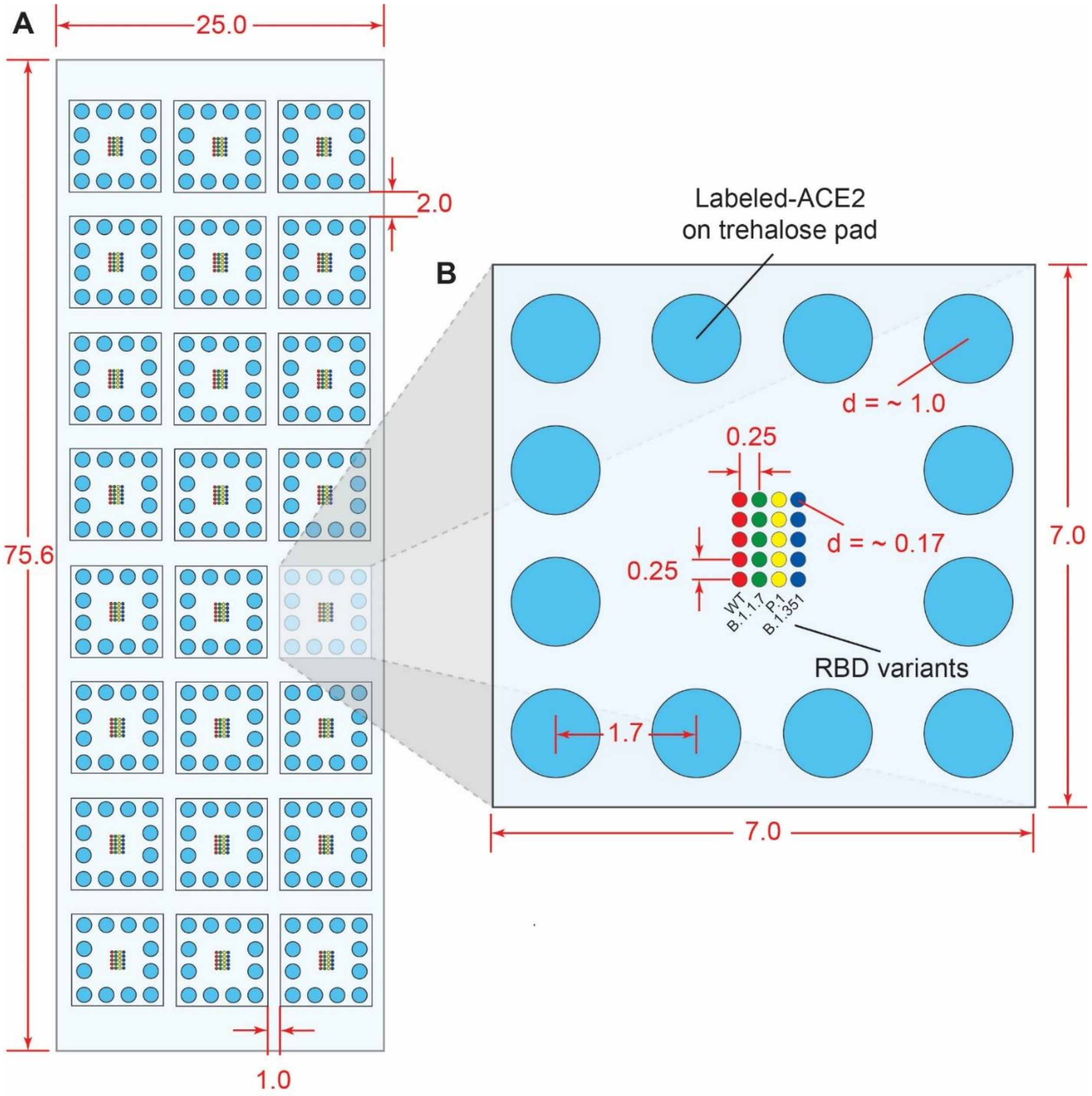

Fig. S1.

CoVariant-SCAN assay print layout. (**A**) Architecture of CoVariant-SCAN assays used in this study. Each standard glass microscope slide contains 24 individual assays. (**B**) Zoomed in view of one individual assay. All measurements listed are in mm.

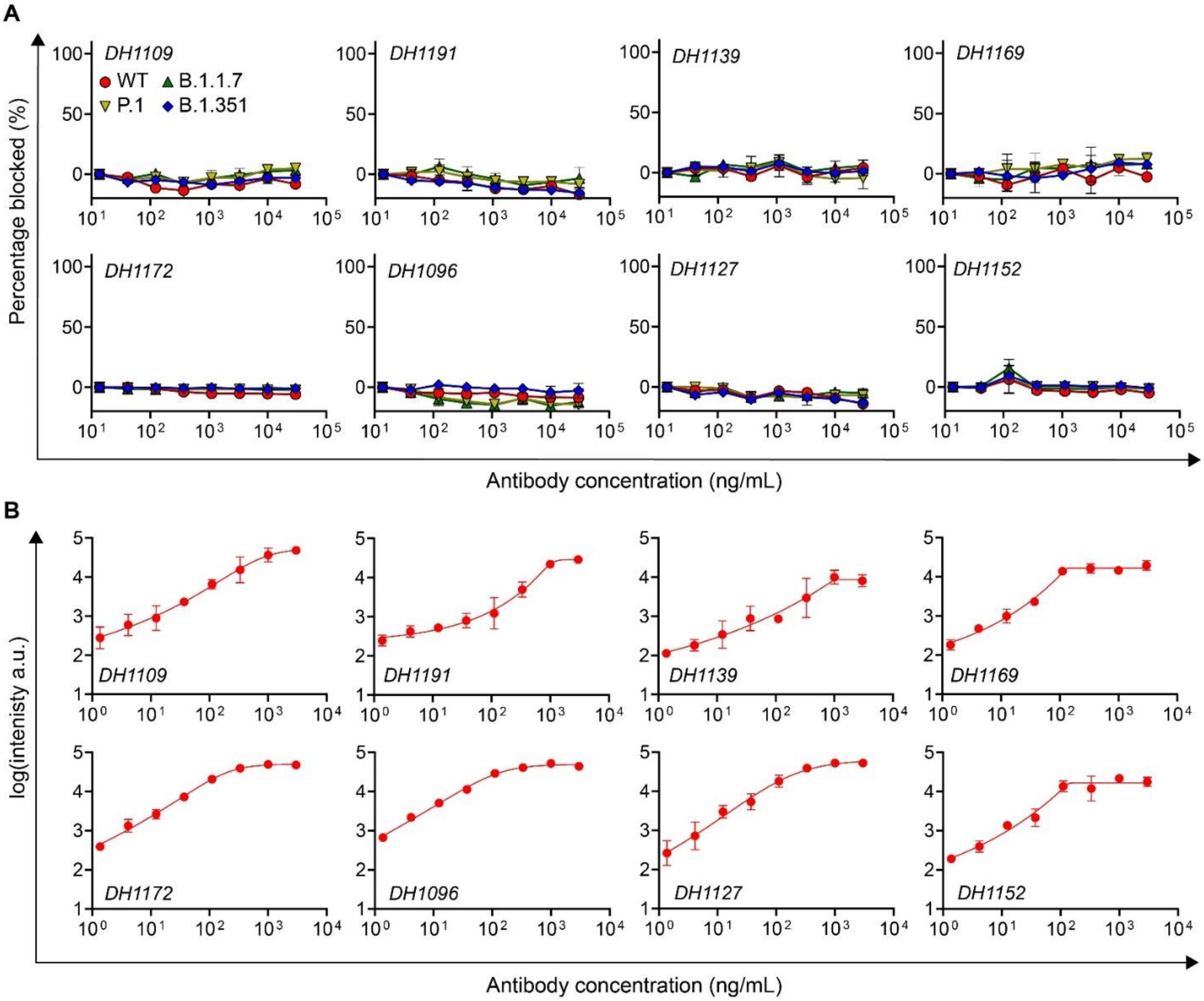

Fig. S2.

Comparison between CoVariant-SCAN and indirect anti-RBD binding assay. (A) Percentage blocked of 8 non-neutralizing mAbs, measured on the CoVariant-SCAN. Each mAb was spiked into undiluted PHS (collected pre-pandemic) and tested at multiple concentrations. Each data point represents the mean of two independent assays, with SD shown. **(B)** The same 8 non-neutralizing antibodies were tested on the anti-RBD indirect assay, as described in the methods. Each mAb was spiked into 1:10 diluted PHS and tested at multiple concentrations. Each data point represents the mean of two independent assays, with SD shown.

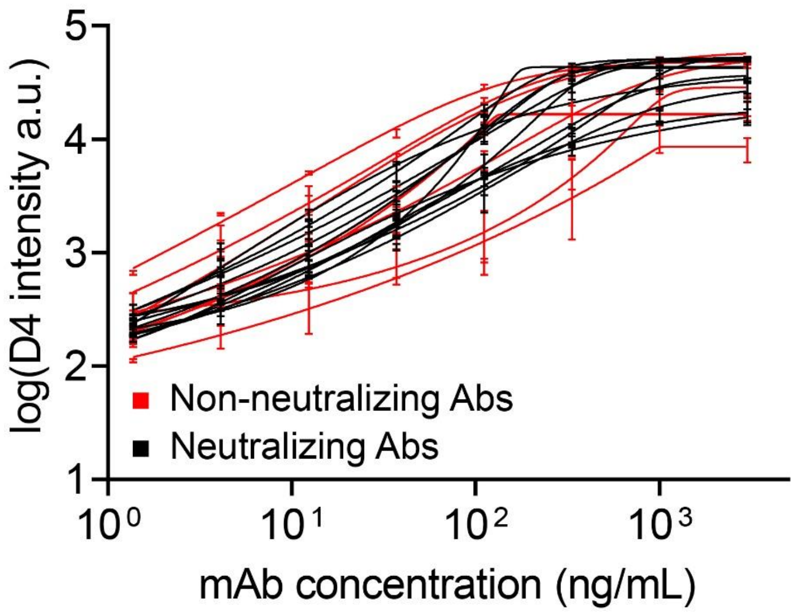

Fig. S3.

Comparison of neutralizing and non-neutralizing mAbs by an indirect assay. All convalescent-patient derived mAbs (neutralizing = black and non-neutralizing = red) were tested by an indirect assay with RBD as the capture antigen. Each mAb was spiked into 1:10 diluted PHS and tested at multiple concentrations. Each data point represents the mean of two independent assays, with SEM shown.

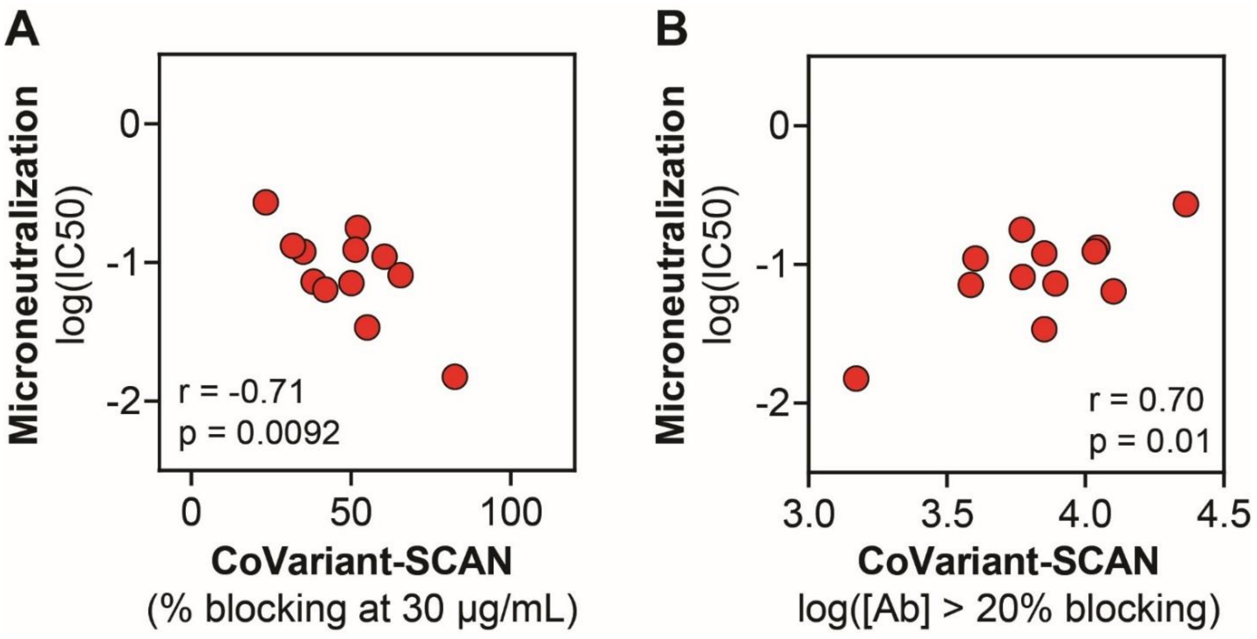

Fig. S4.

Correlation between CoVariant-SCAN and microneutralization IC_{50} for a subset of convalescent patient derived mAbs. (a) Potency of each mAb measured as the percentage blocked at 30 µg/mL on the CoVariant-SCAN versus live virus microneutralization IC_{50}. Correlation is significant (p = 0.0092) with Pearson r = -0.71, which is considered strong. **(b)** Potency of each mAb measured as the inhibitory concentration that blocks > 20% of ACE2 binding versus live virus microneutralization IC_{50}. Correlation is significant (p = 0.01) with Pearson r = 0.70, which is considered strong.

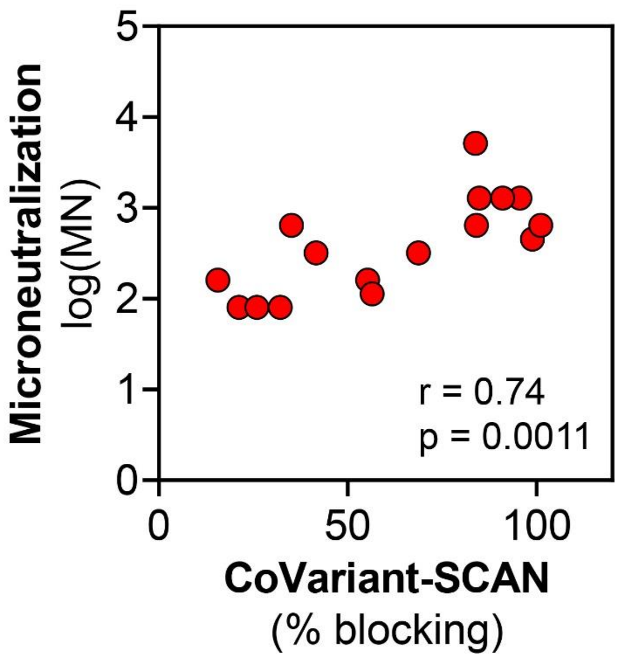

Fig. S5.

Correlation between CoVariant-SCAN and microneutralization for a subset of COVID-19 ICU patients. Microneutralization assays were conducted on a subset of samples from the COVID-19 ICU biorepository. The correlation between both assays is significant ($p = 0.0011$) with a Pearson r correlation of 0.74, which is considered strong.

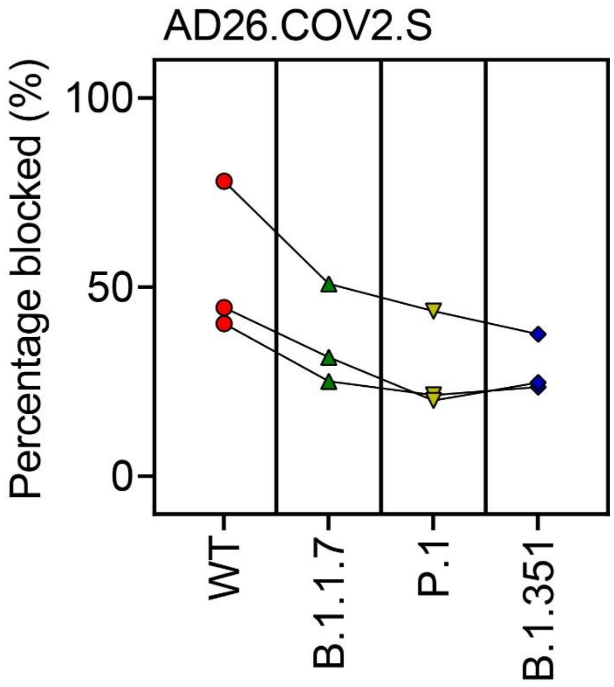

Fig. S6.

CoVariant-SCAN to assess the AD26.COV2.S vaccine. Percent blocking of RBD-ACE2 binding by patient plasma as measured by the CoVariant-SCAN. Data is from patients >2 weeks after receiving the single-shot AD26.COV2.S vaccine, where lines connect data from the same patient.

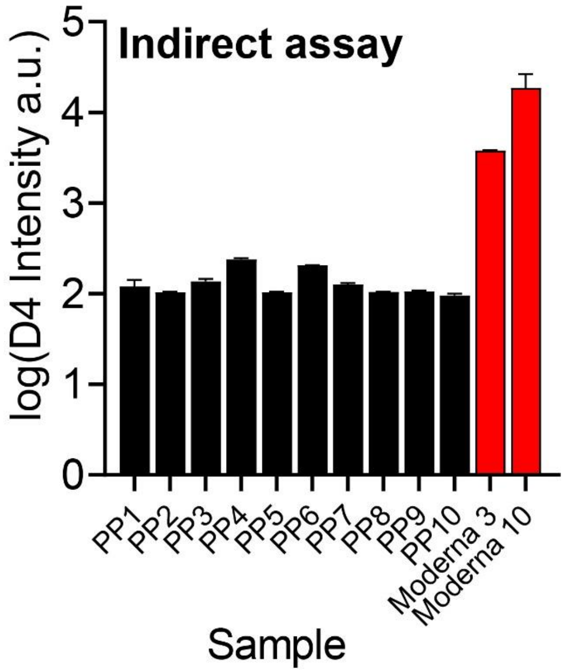

Fig. S7.

Indirect assay for pre-pandemic healthy controls and low nAb Moderna vaccine samples. Pre-pandemic healthy control and two Moderna vaccine plasma samples were tested on the anti-RBD indirect assay, as described in the methods. Each sample was diluted 1:10 in diluent. Each bar represents the mean of two independent assays, with SEM shown.

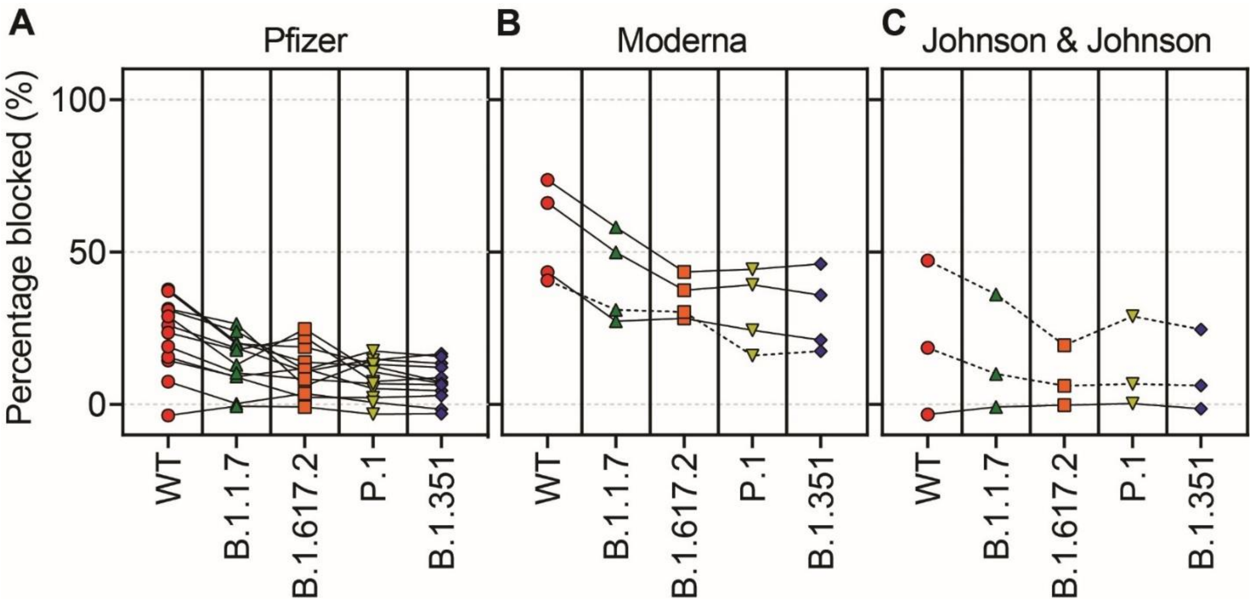

Fig. S8.

Modified CoVariant-SCAN to include B.1.617.2 (Delta) VOC partitioned by vaccine type. Data from Fig. 5C partitioned by vaccine type: (A) Pfizer, (B) Moderna, and (C) Johnson & Johnson. Dashed lines indicate that an individual had a previous confirmed COVID-19 diagnosis.

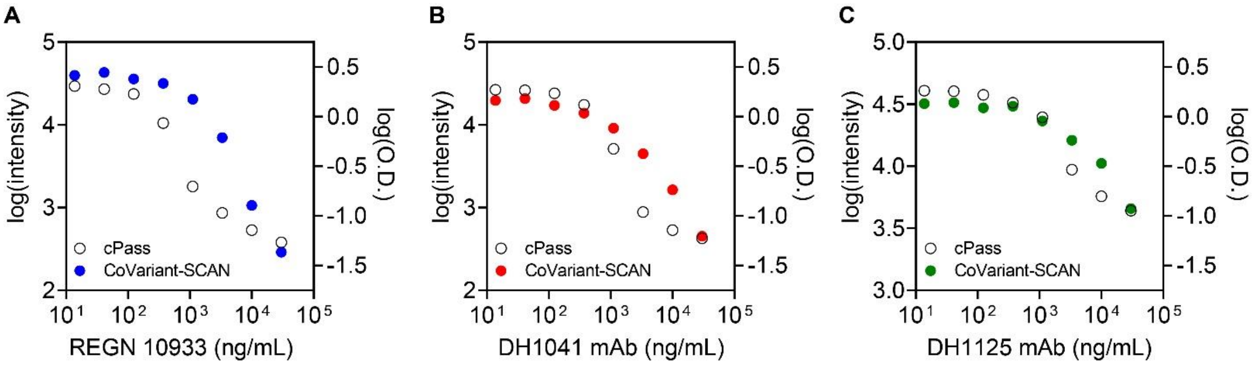

Fig. S9.

Comparison of CoVariant-SCAN and cPass™ SARS-CoV-2 neutralization antibody detection kit. Three mAbs—REGN10933, DH1041, and DH1125—were assayed at multiple concentrations on CoVariant-SCAN (left axis) as described in the materials and methods and cPass™ (right axis). For the cPass™, instructions were followed exactly as outlined in the protocol. Both positive and negative control samples passed the quality control criteria.

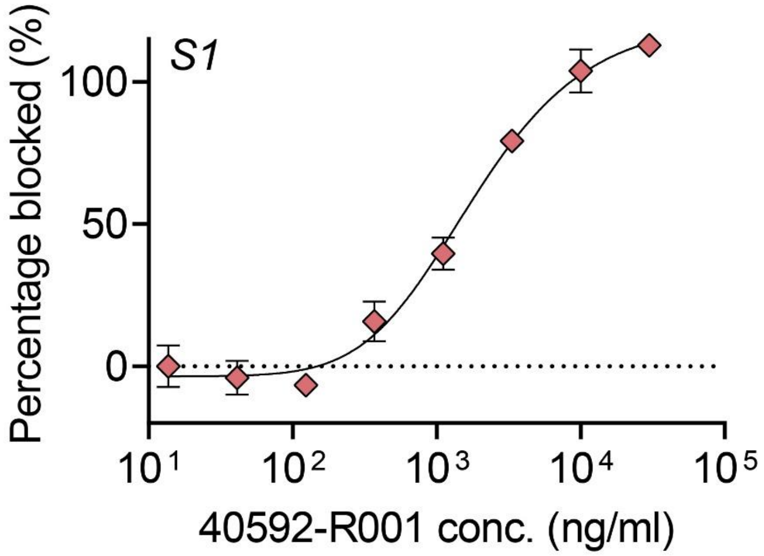

Fig. S10.

Performance of CoVariant-SCAN with S1 as capture antigen showing the percentage of S1-ACE2 binding blocked by 40592-R001 mAb as a function of the mAb concentration. SARS-CoV-2 S1 protein (Sino Biological, cat# 40591-V08H) was printed as a capture antigen at 0.8 mg/mL. mAb was spiked into pre-pandemic PHS, and a 7-point dilution series (30 µg/mL high dose, 1:3 dilutions) was tested in triplicate, with SD shown. Each dose was incubated for 1 h and was then imaged on a GenePix scanner.

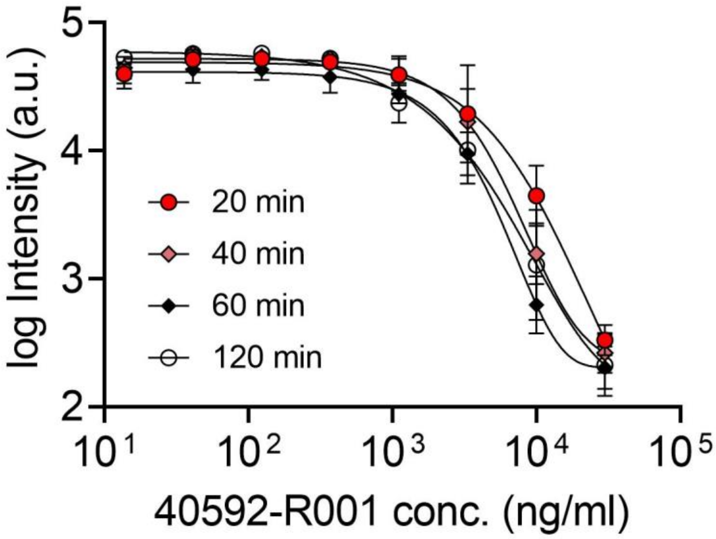

Fig. S11.

Impact of incubation time. We tested a 7-point dose-response curve for mAb 40592-R001 at a starting concentration of 30 µg/mL using WT RBD as the capture antigen at four different incubation times: 20 min, 40 min, 60 min, and 120 min. The log-transformed fluorescent intensity at each dose is plotted against mAb concentration.

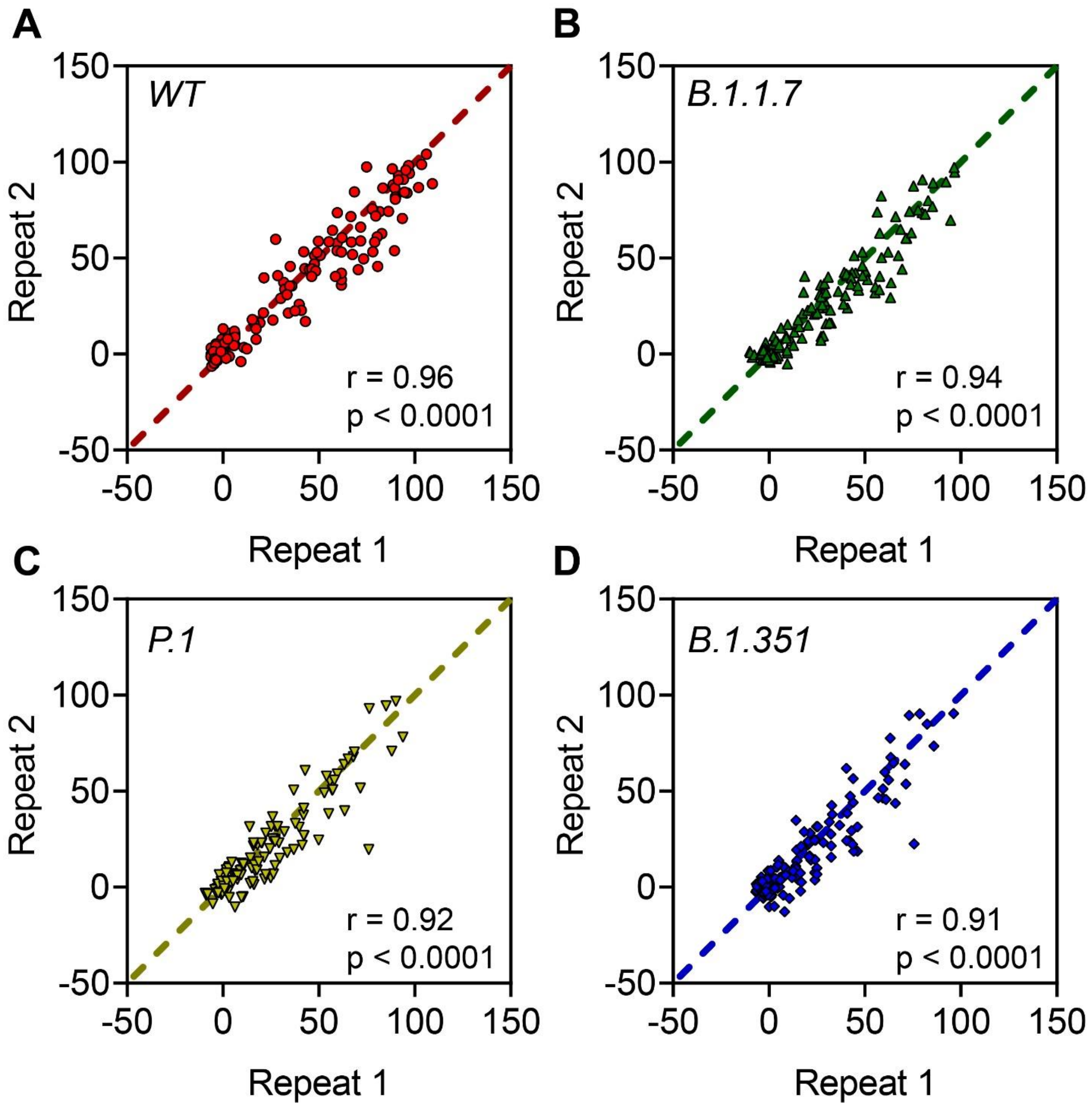

Fig. S12.

Reproducibility of the CoVariant-SCAN. All data presented in Figure 3 and Figure 4 were tested in duplicate. Dashed lines are drawn to have a slope of 1. There is a strong correlation between each repeat for **(A)** WT RBD, **(B)** B.1.1.7 RBD, **(C)** P.1 RBD, and **(D)** B.1.351 RBD, with a Pearson r of 0.96, 0.94, 0.92, and 0.91, respectively ($p < 0.0001$).

Table S1.

Monoclonal antibody summary

Antibody ID	Source	Specificity	Species
DH1143	Convalescent-patient derived	RBD	Human
DH1154	Convalescent-patient derived	RBD	Human
DH1126	Convalescent-patient derived	RBD	Human
DH1179	Convalescent-patient derived	RBD	Human
DH1184	Convalescent-patient derived	RBD	Human
DH1042	Convalescent-patient derived	RBD	Human
DH1043	Convalescent-patient derived	RBD	Human
DH1161.1	Convalescent-patient derived	RBD	Human
DH186	Convalescent-patient derived	RBD	Human
DH1041	Convalescent-patient derived	RBD	Human
DH1047	Convalescent-patient derived	RBD	Human
DH1109	Convalescent-patient derived	RBD	Human
DH1191	Convalescent-patient derived	RBD	Human
DH1139	Convalescent-patient derived	RBD	Human
DH1169	Convalescent-patient derived	RBD	Human
DH1172	Convalescent-patient derived	RBD	Human
DH1096	Convalescent-patient derived	RBD	Human
DH1127	Convalescent-patient derived	RBD	Human
DH1152	Convalescent-patient derived	RBD	Human
40592-MM57	Sino Biological Inc.	RBD	Mouse
SAD-S35	Acro Biosystems	RBD	Human
40592-R001	Sino Biological Inc.	RBD	Rabbit
40591-MM43	Sino Biological Inc.	S1/RBD	Mouse
MAB105802	R&D Systems Inc.	RBD	Mouse
40592-R0004	Sino Biological Inc.	RBD	Rabbit
REGN10933	Regeneron Pharmaceuticals Inc.	RBD	Human
REGN10987	Regeneron Pharmaceuticals Inc.	RBD	Human

Table S2.

Clinical sample summary

Cohort	Number of samples	Age (mean/range)	Gender breakdown (M:F)	Days since symptom onset (mean/range)	Days relative to vaccine 1 (mean/range)
Pre-pandemic (**Figure 3B**)	28	52.8 (17 – 73)	18:10	N/A	N/A
Mild COVID-19 (**Figure 3C**)	18	32.8 (20.1 – 61.4)	10:8	46 (17 – 84)	N/A
Moderate COVID-19 (**Figure 3D**)	18	52.2 (32.1 – 71.3)	7:11	30.2 (24 – 41)	N/A
Severe COVID-19 (**Figure 3E**)	13	54.1 (41 – 66)	7:6	24.9 (17 – 43)	N/A
Pfizer (**Figure 4D**)	18	45.7 (32 – 60.2)	7:11	N/A	38.2 (26 – 69)
Moderna (**Figure 4E**)	23	49.9 (29 – 83.7)	10:13	N/A	44.5 (39 – 77)
Johnson & Johnson (**Figure S5**)	3	43.7 (33.5 – 50.7)	2:1	N/A	15.3 (15 – 16)
Longitudinal samples (**Figure 4B**)	12	40.25 (32 – 48)	9:3	N/A	27.8 (-1 – 77)
Plasma samples from freshly collected blood (**Figure 5C**)	19	32.1 (20 – 52)	10:9	N/A	111.4 (91 – 126)

Data S1. (separate file)

Source data.
